# GYY4137, an H_2_S Slow-Releasing Donor, Prevents Nitrative Stress and α-Synuclein Nitration in an MPTP Mouse Model of Parkinson’s Disease

**DOI:** 10.3389/fphar.2017.00741

**Published:** 2017-10-30

**Authors:** Xiaoou Hou, Yuqing Yuan, Yulan Sheng, Baoshi Yuan, Yali Wang, Jiyue Zheng, Chun-Feng Liu, Xiaohu Zhang, Li-Fang Hu

**Affiliations:** ^1^Institute of Neuroscience, Soochow University, Suzhou, China; ^2^Department of Neurology and Suzhou Clinical Research Center of Neurological Disease, The Second Affiliated Hospital of Soochow University, Suzhou, China; ^3^Department of Pharmacology, School of Pharmacy, Soochow University, Suzhou, China; ^4^Jiangsu Key Laboratory of Translational Research and Therapy for Neuro-Psychiatric-Diseases and College of Pharmaceutical Sciences, Soochow University, Suzhou, China

**Keywords:** GYY4137, hydrogen sulfide, Parkinson’s disease, nitric oxide, α-synuclein nitration

## Abstract

The neuromodulator hydrogen sulfide (H_2_S) was shown to exert neuroprotection in different models of Parkinson’s disease (PD) via its anti-inflammatory and anti-apoptotic properties. In this study, we evaluated the effect of an H_2_S slow-releasing compound GYY4137 (GYY) on a mouse PD model induced by acute injection with 1-methyl-4-phenyl-1,2,3,6-tetrahydropyridine (MPTP). GYY was intraperitoneally (i.p.) injected once daily into male C57BL/6J mice 3 days before and 2 weeks after MPTP (14 mg/kg, four times at 2-h intervals, i.p.) administration. Saline was given as a control. Behavioral tests (rotarod, balance beam, and grid walking) showed that 50 mg/kg GYY significantly ameliorated MPTP-caused motor impairments. At lower doses (12.5 and 25 mg/kg) GYY exhibited a less obvious effect. Consistent with this, immunohistochemistry and western blot analysis demonstrated that 50 mg/kg GYY attenuated the loss of tyrosine hydroxylase (TH) positive neurons in the substantia nigra and the decrease of TH expression in the striatum of MPTP-treated mice. Moreover, at this regimen GYY relieved the nitrative stress, as indicated by the decreases in nitric oxide (NO) generation and neuronal NO synthase (nNOS) upregulation elicited by MPTP in the striatum. The suppression of GYY on nNOS expression was verified *in vitro*, and the results further revealed that Akt activation may participate in the inhibition by GYY on nNOS upregulation. More important, GYY reduced the nitrated modification of α-synuclein, a PD-related protein, in MPTP-induced mice. Overall, our findings suggest that GYY attenuated dopaminergic neuron degeneration and reduced α-synuclein nitration in the midbrain, thus exerting neuroprotection in MPTP-induced mouse model of PD.

## Introduction

Hydrogen sulfide is the third gasotransmitter next to NO and carbon monoxide. It regulates a variety of physiologic and pathologic processes in a wide range of biological systems. For example, the *cse* (a gene encoding the H_2_S synthase CSE) deficient mice developed hypertension early at 7 weeks after birth (Yang et al., 2008). H_2_S also exerted a beneficial role in atherosclerosis and related disorders (Wang et al., 2009). We previously demonstrated its anti-fibrotic property in chronic kidney disease (Song et al., 2014). Indeed, H_2_S was first proposed as an endogenous neuromodulator by Abe and Kimura (1996). However, it was until recently that its function in the central nervous system gains the attention of scientists. Increasing studies identify a potential role of H_2_S in neurodegeneration. For example, sodium hydrosulfide (NaHS, an H_2_S fast-releasing salt) reduced amyloid beta-peptide-induced neuronal injury and ameliorated the learning memory impairment in APP/PS1 transgenic mice (Fan et al., 2013; Liu et al., 2016). In addition, *cse* deficiency contributed to the neurodegeneration in Huntington’s disease (Paul et al., 2014).

Parkinson’s disease is the second most common neurodegenerative disorder, affecting approximately 1.7% of the population over 65 years old. Pathologically, it is featured by dopaminergic neuron losses in the SN and formation of inclusion bodies that are composed of α-synuclein (α-syn). Its etiology remains elusive. Several pathogenic factors such as oxidative stress, mitochondrial dysfunction, protein misfolding and neuroinflammation, have been reported to be involved. In addition, higher level expressions of NO and its synthases were detected in the brains of PD patients and animal models (Eve et al., 1998; Dehmer et al., 2000). Inhibitors or depletion of NOS protected against dopaminergic neuron degeneration in MPTP-induced mouse models (Schulz et al., 1995; Liberatore et al., 1999). These studies strongly suggest a role of nitrative stress in PD progression.

Inhaled H_2_S or NaHS has been shown to exhibit neuroprotection in neurotoxins-induced rodent models of PD (Hu et al., 2010; Kida et al., 2011; Lu et al., 2012). Of note, manipulation of H_2_S gas deals with a lot of safety issues in practice. NaHS is unstable in water solution. Moreover, NaHS or Na_2_S delivers a rapid bolus of H_2_S in aqueous solution within a quite short period (seconds) that does not accurately mimic the biological process of H_2_S release *in vivo*. These limit our understanding to the role of H_2_S in PD pathogenesis. As such, in this study we evaluated the effect of GYY, which slowly releases low but consistent amounts of H_2_S within several hours in aqueous solution at physiologic pH and temperature (Li et al., 2008), in an MPTP-induced mouse model of PD, and the underlying mechanism involved. Our findings clearly demonstrate a protective action of GYY against dopaminergic neuron losses caused by MPTP via its anti-nitrative stress property and inhibition on α-syn nitration, and thus consolidate a favorable role of H_2_S in PD development.

## Materials and Methods

### Animals and MPTP Treatment

Male C57BL/6J mice (8–10 weeks old; 23–28 g weight) were purchased from the SLRC Laboratory (Shanghai, China). The experimental procedures were approved by the Institutional Animal Care and Use Committee (IACUC) of Soochow University. All mice were housed in a SPF animal facility with a temperature at 21–25°C and a controlled light–dark cycle.

Parkinson’s disease animal models were established by intraperitoneally (i.p.) injecting the mice with MPTP (14 mg/kg, Sigma-Aldrich, St. Louis, MO, United States) four times at 2-h intervals. Saline was given as controls. To evaluate the effect of GYY (Cayman Chemical, United States), the mice were administered with GYY at individual doses (12.5, 25, and 50 mg/kg, i.p.) once daily for 3 days prior to MPTP treatment, and continued throughout the experimental period. The animals were sacrificed 2 weeks after MPTP administration for biochemical studies.

### Behavioral Assessment

Behavioral assessments were performed at 2 weeks after MPTP injection. The data were analyzed by independent observers blinded to the treatments.

Rotarod test was performed to estimate motor balance and coordination. Before test, each mouse was trained three times on the rod with an accelerating speed at 5–20 rpm/min. During the test session, the rotational speed remained constant at 18 rpm/min. A trial was stopped if the mouse fell down or the latency to fall reached 5 min. Each mouse was subjected to three trials with an interval of 30 min during the test session, and the average value of the latency to stay on the rod was calculated and included for further analysis.

#### Balance Beam Walking

The mice were placed on a batten and tempted with fodder to cross a wooded balance beam (64 cm long, 1.5 cm wide, 15 cm high). The test was stopped and repeated again if the mouse fell off. The time that a mouse successfully transversed the balance beam was recorded. The test was repeated three times at 5 min intervals, and the mean value of the walking time was presented as the data for analysis. A longer latency to cross the beam indicated a poorer motor coordination.

Grid walking test was applied to evaluate the sensorimotor coordination of hindlimbs in mice. The mice were placed on a 50 ^∗^ 40 cm wire grid with 3 ^∗^ 3 cm grid squares (iron wire at a diameter of 4 mm) in a quiet room with dim lighting, and allowed to walk for 30 s. The number of hindlimb slips was counted by an independent experimenter when the paw completely failed to hold a rung. The animal was put on the grid twice for habituation before test without pre-training. Each trial was repeated three times and the average of foot slips was used for analysis.

### Immunohistochemical Staining

Following the behavioral tests, the animals were deeply anesthetized and perfused with 0.1 M phosphate buffer followed by 4% paraformaldehyde (PFA). Mouse brains were immediately removed from the skull and post-fixed in PFA solution overnight. After that, brains were dehydrated in a serial of alcohol solution and dimethylbenzene was applied to make the brains transparent. Brains were then embedded in paraffin wax. Next, 4 μm thickness sections were cut through SN compacta (SNpc), and every fourth slice was reserved. For staining, sections were deparaffinized and rehydrated, followed by epitope retrieval by boiling the sections in 0.01 M citrate buffer (pH 6.0) for 10–15 min and cooling at room temperature for 20–30 min. After that, sections were thoroughly washed in 0.01 M PBS three times. Endogenous peroxidase activity was blocked with 3% H_2_O_2_ in 0.01 M PBS for 30 min. Next, the sections were incubated in 5% BSA with 0.1% Triton X-100 in PBS for 30 min, followed by incubation with mouse monoclonal anti-TH (1:1000, Sigma, St. Louis, MO, United States) at 4°C overnight. Subsequently, the sections were washed in PBS, incubated with goat anti-mouse secondary antibody at room temperature for 1 h and stained using a DAB kit (Gene Tech, GK500705, China). The sections were observed and photographed using the Zeiss microscope (Carl Zeiss, 37081, Germany).

### Striatal MPP^+^ Level Analysis

Striatal MPP^+^ levels were determined as previously described (Jackson-Lewis and Przedborski, 2007) with minor modifications. In brief, striatum and whole blood were collected 90 min after four injections of MPTP (14 mg/kg, i.p.) at 2-h intervals. The tissue samples were homogenized by sonication in 0.2 M chilled perchloric acid and then centrifuged at 15,000 *g* for 30 min at 4°C. The whole blood samples were kept at room temperature for 30 min and subjected to centrifuged at 1000 *g*, 30 min. Serum was harvested from the resulting supernatant, and mixed with 0.2 M perchloric acid before subjected to the second centrifuged at 15,000 *g* for 30 min at 4°C. The supernatants were transferred into a clean eppendorf tube for MPTP and MPP^+^ analysis.

MPTP/MPP^+^ was analyzed by HPLC equipped with UV detectors (MPTP at 295 nm; MPP^+^ at 245 nm). An aliquot (20 μl) of each supernatant was eluted through a C18 reverse phase column (5 μm, 250 mm × 4.6 mm, SHIMADZU VP-ODS) attached to the HPLC system (Waters, Milford, MA, United States) at a flow rate of 1.0 ml/min. The mobile phase consists of 15% acetonitrile, 50 mM potassium phosphate adjusted to pH 3.2 with ultrapure 18 M phosphoric acid. The detection limit is 3 ng/ml and this method provides good reproducibility.

### NO Production Determination

The striatal NO content was evaluated by measuring the accumulation of nitrite and nitrate using the kit from Jiancheng Bioengineering Institute (Nanjing, Jiangsu, China). Briefly, striatum tissues were homogenized (1:10 m/v) in lysis buffer and centrifuged at 15,000 *g* for 30 min at 4°C. Next, an aliquote (50 μl) of the resulting supernatant was mixed with the dilution buffer at an equal volume, followed by addition with 5 μl nicotinamide adenine dinucleotide phosphate (NADPH), 10 μl flavin adenine dinucleotide (FAD) and 5 μl nitrate reductase and incubation at 37°C for 15 min. After that, 10 μl lactate dehydrogenase (LDH) was added and incubated for another 5 min. Subsequently, 50 μl Griess reagent I (0.1% naphthylethylene diaminedihydrochloride in 1% sulfanilamide) and 50 μl Griess reagent II (2.5% H_3_PO_4_) were added and incubated for 10 min. The optical density of the mixture was measured at 540 nm using a microplate reader (Tecan Infinite M200 PRO, Switzerland). The results were calculated by a standard curve with sodium nitrite, and expressed as μmol per gram protein. The protein concentrations of striatal homogenates were determined using the BCA protein assay kit (Thermoscientific, United States).

### Western Blot Analysis

Whole striatum tissues were homogenized with lysis buffer and centrifuged at 13,200 rpm for 20 min at 4°C. Protein concentrations of the homogenates were determined using the BCA protein assay kit. Equal amounts of total proteins were loaded in 8–12% sodium dodecyl sulfate polyacrylamide gels and then transferred onto PVDF membranes. Membranes were incubated with specific antibodies: anti-TH (1:2000, Sigma, T1299, United States), anti-nNOS (1:1000, BD, 610308, New York, United States), anti-nitrated α-syn (1:750, Thermo Scientific, MA5-16142, United States), anti-α-syn (1:500, CST, 2642, United States), anti-p-Akt (1:500, CST, 4060s, United States), anti-Akt (1:1000, CST, 4691s, United States), and anti-β-actin (1:5000, Sigma, A3584, United States) at 4°C overnight. Blots were then briefly washed with PBS followed by incubation with the corresponding secondary antibodies (Jackson Lab, United States) for 1 h at room temperature. Protein densities were detected using an enhanced chemiluminescence kit and analyzed by ImageJ software.

### Cell Culture and Treatment

PC12 cells were purchased from the Institute of Cell Biology, Chinese Academy of Sciences (China), and cultured in RPMI1640 medium with 10% fetal bovine serum and 1% penicillin/streptomycin. The cultures were maintained in a incubator with 5% CO_2_/95% air at 37°C. For experimentation, cells were seeded into a 12-well plate and cultured overnight before treatment.

### Statistical Analysis

All data were presented as mean ± SEM of at least three independent experiments. All statistical analyses were performed with GraphPad Prism 5.0 software. The differences among groups were analyzed using one-way analysis of variance (ANOVA) followed by *Tukey’s post hoc* analysis. Statistical significance was set at *P* < 0.05.

## Results

### GYY Improves Motor Deficits Induced by MPTP in Mouse

**FIGURE 1:**
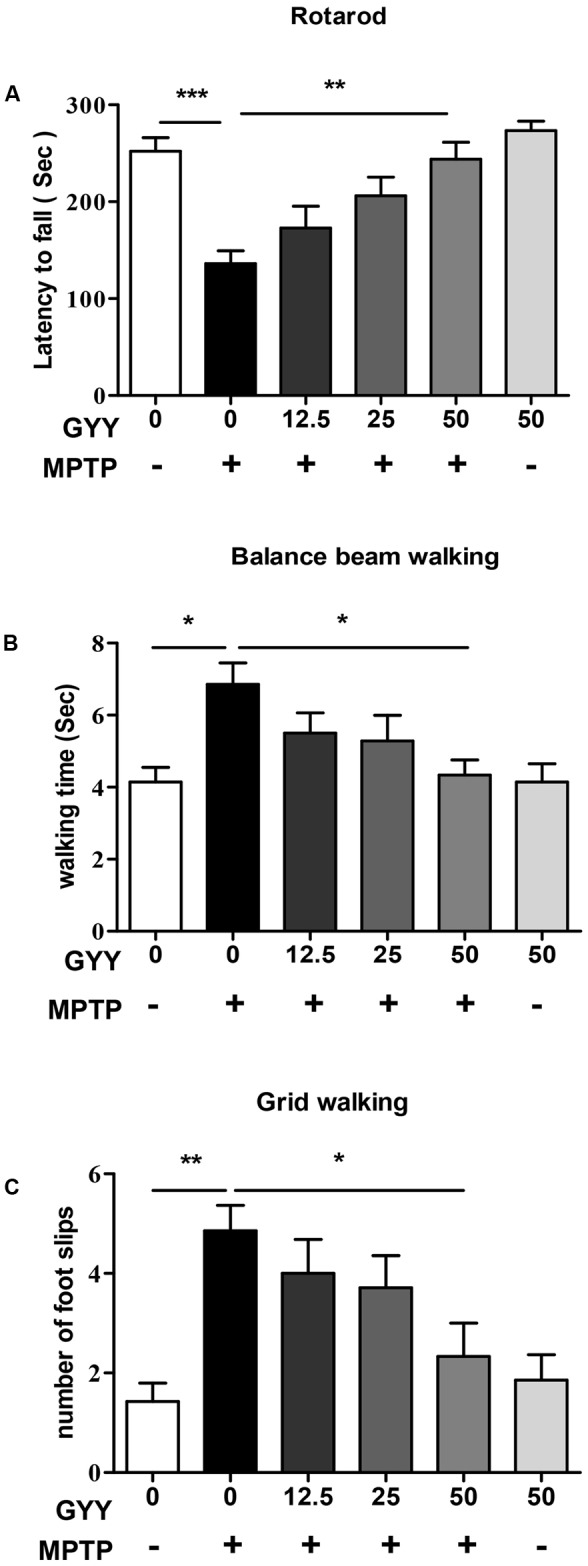
GYY ameliorated motor deficits in MPTP-induced mouse model of PD. Performance on the rotarod **(A)** and balance beam **(B)** was impaired by MPTP administration, as indicated by a shorter latency to stay on the rod **(A)** and a longer period to cross the beam **(B)** in MPTP-injected mice. The number of foot slips in the hindpaw **(C)** was also increased by MPTP injection compared to controls. GYY (12.5, 25, and 50 mg/kg/d, i.p.), administered 3 days before and 2 weeks after MPTP injection, alleviated the MPTP-induced motor dysfunction to a different extent, and the effect of 50 mg/kg GYY was consistently significant. Treatment with 50 mg/kg GYY or saline alone did not affect the motor behavior. Results are presented as mean ± SEM from 6 to 7 mice per group. ^∗^*P* < 0.05, ^∗∗^*P* < 0.01, ^∗∗∗^*P* < 0.001; one-way ANOVA.

To study the ability of GYY to affect MPTP-induced impairment in motor function, we assessed the animals’ motor coordination using rotarod, balance beam walking, and grid walking test at 2 weeks after MPTP injection. The results are shown in **Figure 1**. Compared to saline-treated group (control), the latency to fall off the rod significantly decreased in MPTP-injected mice (**Figure 1A**). Co-treatment with GYY at 25 and 50 mg/kg prolonged the time of staying on the rod compared to MPTP-only group. Balance beam walking test also showed that 50 mg/kg GYY co-treatment improved the MPTP-induced motor deficits, as indicated by a shorter time period to cross the beam compared to MPTP only group (**Figure 1B**). Similarly, in grid walking test we found that MPTP injection increased the number of foot slips in mice, implying that the sensorimotor function was also impaired by MPTP. This was markedly attenuated by 50 mg/kg GYY co-treatment (**Figure 1C**). And the treatment with GYY alone at 50 mg/kg, the maximal tested dose in this study, did not have any impact on the motor behavior.

### Striatal MPP^+^ Levels Are not Affected by GYY Preventive Treatment

**FIGURE 2:**
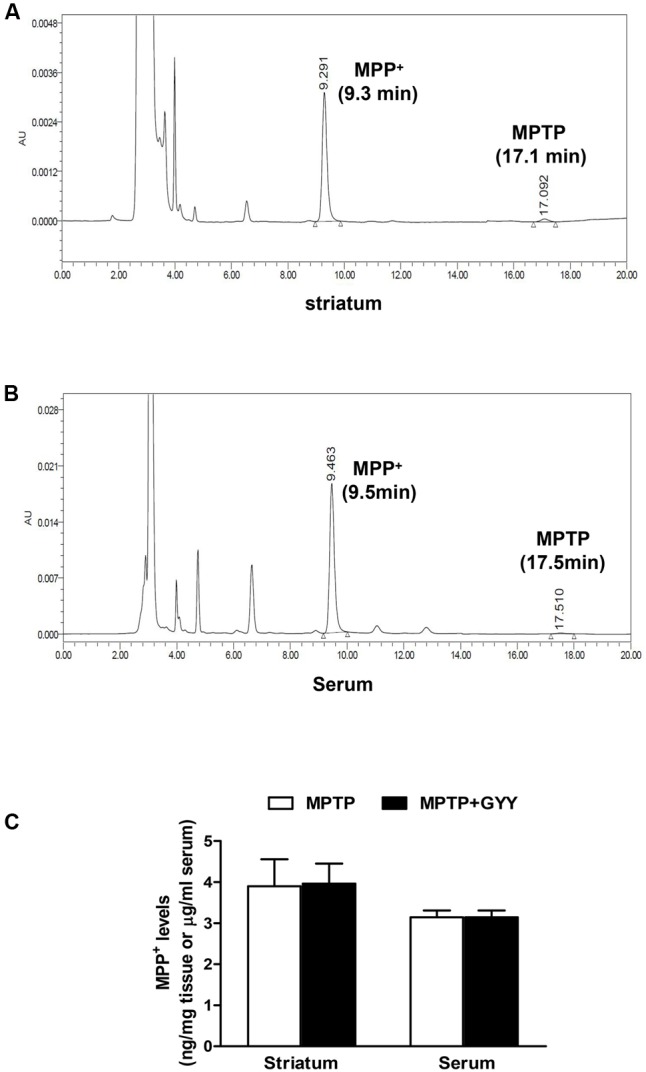
GYY did not affect MPTP metabolism. Striatal and serum MPP^+^ levels quantification by HPLC in combination with UV detection. Panels show typical chromatographs of striatal **(A)** and serum **(B)** MPP^+^ and MPTP. Typical retention times for MPP^+^ at 9.3 min and MPTP at 17.5 min. MPTP levels, almost near the detection limit (3 ng/ml), were much lower than those of MPP^+^ and thus were not included for analysis. Mice received four injections of MPTP (14 mg/kg, i.p., at 2 h intervals), with or without GYY (50 mg/kg, i.p.) pretreatment for 3 days (five mice per group). MPTP was given at 1 h after the last GYY injection. Striatum and whole blood were harvested 90 min after the last MPTP administration. GYY pretreatment did not affect the striatal and serum MPP^+^ levels resulting from MPTP metabolism **(C)**. Student’s *t*-test. *N* = 5 for serum sample; *n* = 10 for striatal homogenates (both sides of the striatum from five mice per group).

As MPTP-caused neurotoxicity to dopaminergic neurons is dependent on the metabolism of MPTP to its active metabolite MPP^+^ via monoamine oxidase (MAO) in the nigrostriatal system, the effect of GYY on striatal MPP^+^ levels was determined in a parallel study. In this part, 10 male C57BL/6J mice (10 weeks old) were applied and randomly divided into two groups: MPTP and GYY + MPTP group (*n* = 5 per group). For MPTP group, the mice received four injections with MPTP (14 mg/kg, i.p., at 2 h intervals). For GYY + MPTP group, the mice were treated with GYY (50 mg/kg, i.p.) once daily for 3 days followed by MPTP administration at 1 h after the last injection with GYY. No significant changes were observed in the striatal MPP^+^ level in MPTP-treated mice with and without GYY (50 mg/kg, i.p.) pretreatment (MPTP 3.90 ± 1.98 nmol/mg tissue; GYY + MPTP group, 3.96 ± 1.46 nmol/mg tissue; *P >* 0.05; **Figure 2**), which was measured 90 min after the last MPTP injection. Measurement of serum MPP^+^ levels revealed no significant difference between the two tested groups either (MPTP 3.25 ± 0.39 μg/ml; GYY + MPTP group, 3.14 ± 0.33 μg/ml, *P* > 0.05). This indicates that the observed effects of GYY on MPTP-induced neurotoxicity are not caused by the reduction of MPTP conversion into MPP^+^.

### GYY Attenuates Dopaminergic Neuron Losses in the Substantia Nigra

**FIGURE 3:**
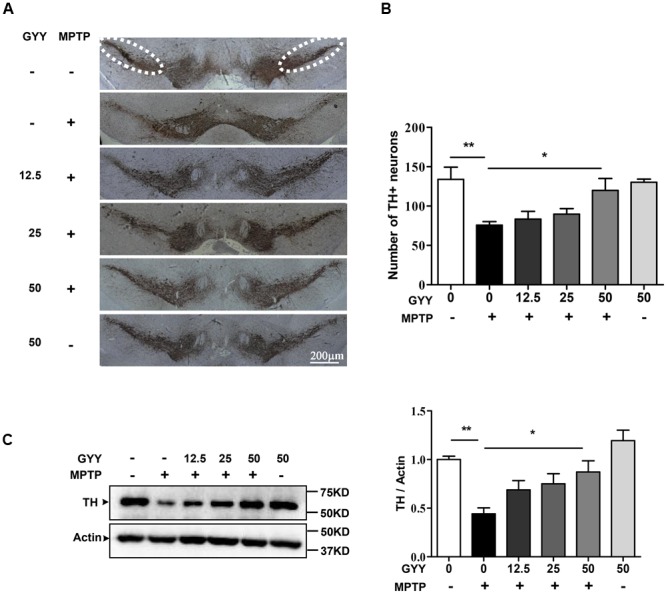
GYY attenuated dopaminergic neuron losses in MPTP-treated mice. **(A)** MPTP injection caused a reduction in the number of TH positive neurons in the SNpc (the circled area in dotted line). Group data was shown in **(B)**. The number of TH positive neurons in both sides was counted from six brain slices for each mouse, with 3–5 mouse brains per group. The counting was performed by the experimenters blind to the treatments. **(C)** Striatal TH expression in MPTP-intoxicated mice was markedly reduced. *N* = 6 per group. Co-treatment with 50 mg/kg GYY was able to attenuate the loss of TH neuron in the SNpc **(A,B)** and the decrease of TH expression **(C)** in the striatum. The molecular masses of the protein markers were indicated to the right of the blots. ^∗^*P* < 0.05, ^∗∗^*P* < 0.01, ^∗∗∗^*P* < 0.001; one-way ANOVA.

The dopaminergic neuron degeneration was examined by quantifying the number of TH immunoreactive neurons in the SNpc using immunohistochemistry study. The result showed that MPTP caused an approximate 40% decrease in the number of dopaminergic neurons in the SN compared to saline, and this was attenuated in the mice co-treated with 50 mg/kg GYY (**Figures 3A,B**). Treatment with GYY alone did not have any impact on TH-positive staining in the SN. Western blot analysis showed that 50 mg/kg GYY co-treatment prevented the reduction of striatal TH protein level caused by MPTP (**Figure 3C**). These results indicate that co-treatment with GYY at a suitable dose was able to prevent dopaminergic neuron losses in the SN of MPTP-treated mice.

### GYY Mitigates MPTP-Induced NO Overproduction and Nitrated Modification of α-Synuclein in the Striatum

**FIGURE 4:**
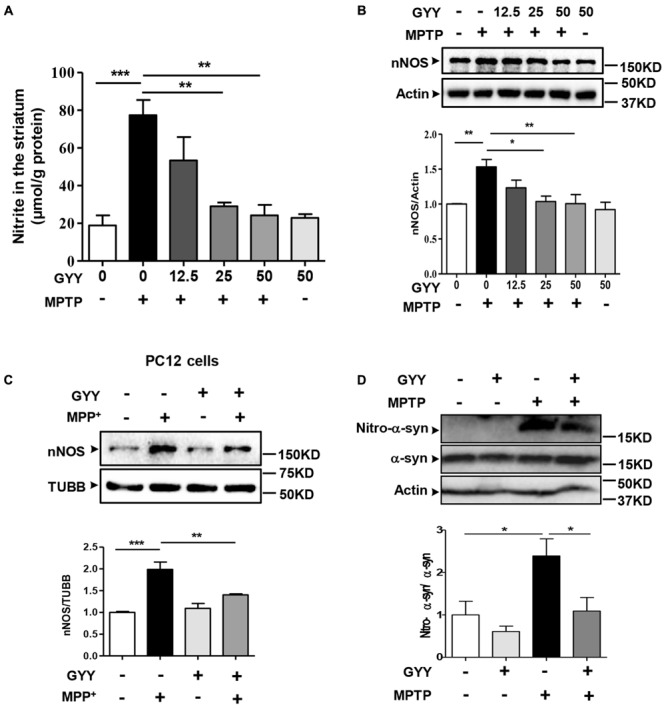
GYY mitigated MPTP-induced nitrative stress and α-syn nitration in the striatum. **(A)** Co-treatment with 50 mg/kg GYY abolished NO overproduction in the striatum of MPTP-injected mice. The nitrite level was normalized by the protein concentration of striatal homogenates, *n* = 3. **(B)** Western blot study showed an upregulation of striatal nNOS induced by MPTP compared to controls, and this was attenuated by GYY (25, 50 mg/kg) co-treatment. *N* = 5 per group. Treatment with 50 mg/kg GYY alone did not affect NO generation or nNOS expression. **(C)** Effect of GYY on nNOS expressions *in* PC12 cells treated with 100 μM MPP^+^ or vehicle for 24 h. Cells were pretreated with 100 μM GYY for 1 h before MPP^+^ being added. Relative values were obtained after normalization to loading controls (Tubulin/TUBB), *n* = 4. **(D)** Co-treatment with 50 mg/kg GYY led to a reduced nitrated modification of α-syn, but not the total α-syn in the striatum, indicated by a lower ratio of nitro-α-syn over total α-syn compared to MPTP-treated mice. Actin served as loading controls. Mean ± SEM, *n* = 5. ^∗^*P* < 0.05, ^∗∗^*P* < 0.01, ^∗∗∗^*P* < 0.001; one-way ANOVA.

Overproduction of NO and its associated nitrative stress participate in PD pathology (Jimenez-Jimenez et al., 2016). In line with previous studies (Dehmer et al., 2000), we observed an accumulation of nitrite, a metabolite of NO, in the striatum of the mice receiving MPTP administration (**Figure 4A**). This increase of NO generation was abated in the mice co-treated with GYY at the dose of 25 and 50 mg/kg, respectively. Likewise, co-treatment with GYY at this regimen inhibited the MPTP-induced upregulation of nNOS expression in the striatum (**Figure 4B**). Treatment with GYY alone did not modulate NO or nNOS expression level. The result was verified by an *in vitro* study, which demonstrated that pre-treatment with 100 μM GYY prevented MPP^+^ (100 μM, 24 h) elicited increase of nNOS expression in PC12 cells (**Figure 4C**).

Excessive NO generation often results in peroxynitrite formation and tyrosine nitration in proteins. Human α-syn contains four tyrosine residues (Tyr39, Tyr125, Tyr133, and Tyr136) that can be nitrated (Schildknecht et al., 2013), which may play a role in protein aggregation in PD. Therefore, we further examined the effect of GYY on α-syn nitration by western blot using the specific antibody against nitrated α-syn (Tyr125, Tyr133). The result showed that although the total α-syn level was not altered in the striatum following MPTP injection, the nitrated α-syn level was markedly elevated in MPTP-treated mice, and this elevation was abolished by GYY co-treatment (**Figure 4D**).

### Akt Signaling Mediates GYY-Induced Inhibition on nNOS Upregulation

**FIGURE 5:**
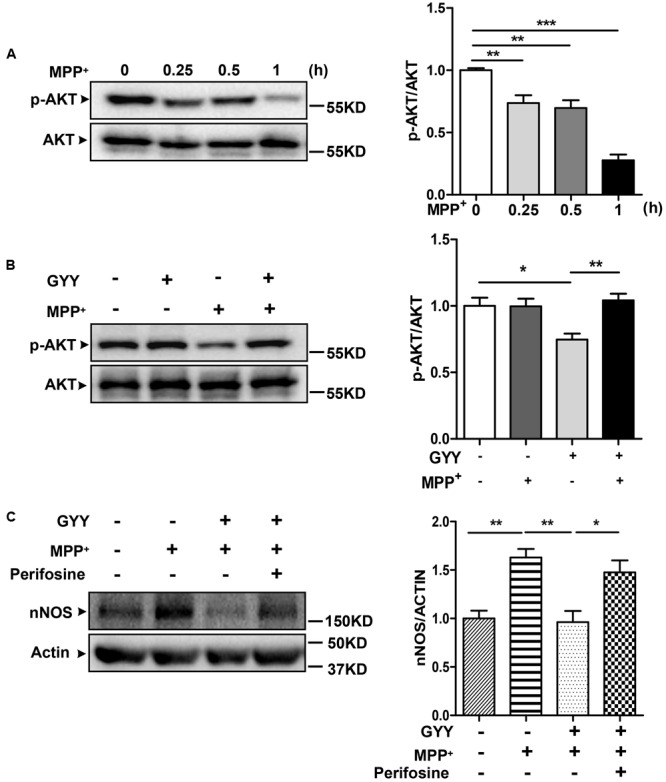
Akt activation was involved in the inhibition by GYY on nNOS expression. **(A)** Decreases of AKT phosphorylation at different time points after 100 μM MPP^+^ treatment in PC12 cells, *n* = 3. **(B)** Pretreatment with 100 μM GYY for 1 h was found to reverse the decrease of AKT phosphorylation caused by MPP^+^ in cells. *N* = 6. **(C)** The inhibition of GYY pretreatment on nNOS expression was abolished in the presence of AKT inhibitor perifosine. PC12 cells were pretreated with perifosine (5 μM) for 1 h, followed by incubation with GYY for 1 h and subsequent exposure to MPP^+^ for 24 h. *N* = 5. *^∗^P* < 0.05, ^∗∗^*P* < 0.01, ^∗∗∗^*P* < 0.001; one-way ANOVA.

Last, we studied the signaling pathway that underlies the inhibition by GYY on nNOS expression. Treatment with 100 μM MPP^+^ was found to reduce the phosphorylation of Akt Ser 473 in a time-dependent manner, which was detected early at 0.25 h and remained to be dramatically declined at 1 h after treatment in PC12 cells (**Figure 5A**). Pretreatment with 100 μM GYY almost reversed the decline of Akt phosphorylation caused by MPP^+^ exposure (**Figure 5B**). However, co-treatment with Akt inhibitor perifosine (5 μM) was able to attenuate the GYY-mediated inhibition on nNOS expression in MPP^+^-treated cells (**Figure 5C**). These results indicate that Akt signaling may be activated and thus contribute to the inhibition on nNOS expression by GYY.

## Discussion

In this study we demonstrate the potential of GYY as a neuroprotectant against dopaminergic neuron losses in a mouse model of PD based on the following findings. First, intraperitoneal injection with GYY at an appropriate dose (50 mg/kg) alleviated MPTP-induced motor impairments in mice. Second, this H_2_S slow-releasing compound attenuated dopaminergic neuron losses in the SNpc caused by MPTP. Last, GYY mitigated MPTP-induced nitrative stress and α-syn nitration *in vivo*. In addition, GYY preventive treatment did not affect MPTP metabolism, as serum and striatal MPP^+^ levels in GYY + MPTP group did not differ from those in MPTP group. To our knowledge, this is the first study demonstrating the neuroprotection of an H_2_S slow-releasing compound in the MPTP-induced mouse model of PD.

Hydrogen sulfide is linked to the pathogenesis of PD. Hu et al. (2010) first reported that NaHS exerted neuroprotection against dopaminergic neuron losses in 6-hydroxydopamine and rotenone-induced rat models of PD. This beneficial role of H_2_S in PD was subsequently confirmed by other studies. Two independent groups demonstrated that systemic administration with NaHS or inhaled H_2_S prevented neurodegeneration and movement dysfunction in the MPTP-induced mouse model of PD (Kida et al., 2011; Paul et al., 2014). Vandiver et al. (2013) demonstrated that sulfhydration, a post-translational modification afforded by H_2_S, mediated the neuroprotective actions of parkin, and this study again highlighted a potential role of H_2_S in PD. However, it should be noted that most studies utilized NaHS as an H_2_S donor, which often leads to a burst of H_2_S release in solution. This does not mimick the biological synthesis of H_2_S, which is tightly regulated *in vivo*. To fully understand the role of H_2_S in PD, it is essential to assess the effect of H_2_S slow-releasing compounds in PD models. GYY is a water-soluable compound that releases H_2_S slowly over a period of hours *in vitro and in vivo*, producing H_2_S-related beneficial effects in several pathological conditions (Lin et al., 2016; Weber et al., 2017). Thus, in this study we evaluated the impact of GYY on a classic mouse model of PD induced by acute MPTP injection. Functionally, administration of GYY improved the motor impairments. Pathologically, GYY co-treatment attenuated the loss of dopaminergic neurons in the SN. Moreover, GYY pretreatment did not alter the striatal MPP^+^ level, which excluded the possibility that GYY inhibited the MPTP metabolism via MAO although sulfides was reported to be MAO inhibitors (Warenycia et al., 1989; Stewart et al., 2014). These findings suggest the protective effects of GYY in this PD model. In support of this, two recent articles published in *Movement Disorders* proposed that the action of coffee consumption and smoking in lowering the risk of PD may be linked to H_2_S. Coffee intake was shown to improve the Prevotella population, which served as an important source of H_2_S generation and was decreased in the gut of PD patients (Cakmak, 2015, 2016; Scheperjans et al., 2015). But, it remains to delineate whether the role of H_2_S in PD relates to the gut microbiota.

Human α-syn contains four tyrosine residues, which can be nitrated by peroxynitrite when excessive NO is generated. 3-nitrotyrosine, as a footprint of tyrosine nitration in proteins, was detected in both PD patients and neurotoxins-induced animal models of PD (Schulz et al., 1995; Good et al., 1998). Moreover, several studies demonstrate that lewy bodies, the pathologic hallmark of PD and other synucleinopathies, largely contain α-syn that is modified by nitration on tyrosine residues (Giasson et al., 2000). So far, the pathophysiological function of nitrated α-syn remains elusive. Some studies report that α-syn nitration may facilitate protein aggregates formation and thus contribute to the pathogenic processes of PD and other synucleinopathies. Nitrated α-syn was suggested to serve as a clinical biomarker for PD diagnosis (Fernandez et al., 2013). In this study, we found that MPTP caused an increase of nitrated but not the total α-syn level in the striatum, and this increase was attenuated by GYY co-treatment. This also indicates the inhibition by GYY on nitrative stress induced by MPTP.

As a sibling of NO, H_2_S has been demonstrated to directly and indirectly interact with NO in biological systems. Moreover, H_2_S signaling, mediated by *S*-sulfhydration of its targeting proteins, often functions in an opposite manner to NO (Mustafa et al., 2009; Lu et al., 2013). In this study, GYY not only reduced the excessive generation of NO but also blocked the increase of nNOS expression induced by MPTP in the striatum. However, treatment with GYY did not have an obvious impact on NO or nNOS expression in control mice. This may be due to the relative lower level of NO under physiological conditions. Alternatively, the slower release of H_2_S from GYY which results in a consistent but lower level of H_2_S may also explain the lack of a direct effect of GYY on NO and nNOS expression, as previously reported (Li et al., 2008). Here, we demonstrated an indirect effect of GYY on nNOS expression, which may, at least in part, be mediated by Akt signaling, because GYY was found to enhance Akt activation. And in the presence of the Akt inhibitor perifosine, GYY-induced inhibition on nNOS expression was obviously abated. Therefore, activation of Akt signaling may participate in the suppression on nitrative stress and the neuroprotection by GYY on this mouse model. However, it should be cautious that a number of treatments have been shown to be efficacious in the animal models but finally failed in human clinical trials. Therefore, much more preclinical investigations need to be carried out to fully understand the role of H_2_S and its related strategies for treatment in PD.

In summary, our data demonstrated that GYY, a novel H_2_S slow-releasing compound, was protective in an *in vivo* mouse model of PD via its inhibition on nitrative stress. The findings may have implications for the research and development of H_2_S-based therapies for PD.

## Ethics Statement

This study was carried out in accordance with the recommendations of the Laboratory Animal Care Guidelines of the Institutional Animal Care and Use Committee (IACUC) of Soochow University. The protocol was approved by the Institutional Animal Care and Use Committee (IACUC) of Soochow University.

## Author Contributions

XH: study design, data acquisition, data analysis and manuscript preparation. YY and YS: experiments design, sample collect and data analysis. BY, YW, and JZ: sample acquisition, data collection, statistical analysis. C-FL and XZ: study design and manuscript revision. L-FH determined the study theme, designed the experiments, ensured all the data and approved the final version to be published. All authors have contributed to this article.

## Conflict of Interest Statement

The authors declare that the research was conducted in the absence of any commercial or financial relationships that could be construed as a potential conflict of interest.

## Abbreviations

CSE: cystathionine-γ-lyase
GYY: GYY4137
HPLC: high-performance liquid chromatography
H_2_S: hydrogen sulfide
i.p.: intraperitoneal
MPTP: 1-methyl-4-phenyl-1,2,3,6-tetrahydropyridine
NO: nitric oxide
NOS: NO synthase
PD: Parkinson’s disease
SN: substantia nigra
α-syn: α-synuclein
TH: tyrosine hydroxylase
